# Temporal trends in late-life depressive symptom trajectories: a longitudinal population-based cohort study from Stockholm, Sweden

**DOI:** 10.1136/bmjph-2026-005098

**Published:** 2026-07-22

**Authors:** Franziska Steffens, Federico Triolo, Neda Agahi, Stefan Fors, Lena Dahlberg, Caterina Gregorio, Davide Liborio Vetrano, Serhiy Dekhtyar

**Affiliations:** 1 Aging Research Center, Department of Neurobiology, Care Sciences and Society, Karolinska Institutet and Stockholm University, Stockholm, Sweden; 2 Vrije Universiteit, Department of Psychiatry, Amsterdam UMC, Amsterdam, Netherlands; 3 Region Stockholm, Center for Epidemiology and Community Medicine, Stockholm, Sweden; 4 Dalarna University, School of Health and Welfare, Falun, Sweden; 5 Stockholm Gerontology Research Center, Stockholm, Sweden

**Keywords:** Depression, trends, Age Factors, Epidemiology

## Abstract

**Background:**

While studies of depression prevalence in Sweden suggest improving trends over time among older adults, longitudinal approaches can provide a more nuanced perspective on the changing burden of late-life depression. This study mapped 19-year trajectories of depressive symptoms among cohorts of older adults born between 1904 and 1951 residing in Stockholm, Sweden.

**Methods:**

We examined 2960 individuals aged 60–99 years from the ongoing longitudinal population-based Swedish National Study on Aging and Care in Kungsholmen (SNAC-K) representing eight partially overlapping birth cohorts followed up between 2001 and 2020. Depressive symptoms were measured using the Montgomery-Åsberg Depression Rating Scale. Linear mixed-effect models with cubic splines were used to derive depressive symptom trajectories across age and birth cohort. Baseline education, functional impairment and loneliness were considered as potential explanatory factors for any temporal patterns.

**Results:**

Depressive symptoms increased with age in all birth cohorts. Age-related symptom increase accelerated in each later-born cohort (eg., at age 84, predicted MADRS was 2.9 (95% CI 2.6 to 3.2) in the birth cohort 1920–1924, 3.2 (95% CI 3.0 to 3.5) in the birth cohort 1929–1933 and 4.0 (95% CI 3.8 to 4.3) in the birth cohort 1935–1939). Among similarly aged individuals separated in time, a worsening burden over time was noted among the 60-year-olds but not the 81-year-olds. These patterns persisted after considering baseline education, functional impairment and loneliness.

**Conclusions:**

Despite some suggestions of declining prevalence, when considered longitudinally, late-life depressive burden appears to be increasing more recently in this Stockholm-based population. Given the growing number of older adults in Sweden, these trends carry important public health implications for prevention and management of depression in late life.

**WHAT IS ALREADY KNOWN ON THIS TOPIC**

**WHAT THIS STUDY ADDS**

- Using longitudinal data with up to 19 years of follow-up, this study shows a steeper age-related increase of depressive symptoms in later later-born cohorts from Stockholm, Sweden.

**HOW THIS STUDY MIGHT AFFECT RESEARCH, PRACTICE OR POLICY**

- This study emphasises the value of longitudinal evidence for monitoring time trends and guiding mental healthcare planning.

## Introduction

 As a leading cause of disability, depression is a major public health concern.^1^ Worldwide, an estimated 4% were living with a depressive disorder in 2021.^1^ As both global and Swedish populations continue to age, late-life depression is likely to become especially burdensome. Cross-sectional studies of late-life depression from Sweden indicate a prevalence of 5–15% for any type of depression and 1–5% for severe depression, with variation due to age group, year of assessment, or region of study.^2^ The few studies exploring temporal changes in the burden of late-life depression in Sweden have noted both increasing and declining prevalence over time. For example, a repeated cross-sectional analysis, using population-based data, found that the prevalence of clinical depression had increased between 1976 and 2006 among 75-year-olds from Gothenburg, as well as between 2000 and 2015 among 85+-year-olds from Northern Sweden.^3,4^ However, re-analyses of the Gothenburg data incorporating recent assessment waves found that depression prevalence had declined in 2014–2017 among both 85-year-olds and 70-year-olds (the latter among women only).^5,6^ An updated assessment of late-life depression in Sweden, using data from other Swedish regions, alongside a broader age-range perspective that also considers younger-olds (ie, starting from age 60), is warranted. This will provide important new insights for predicting future depression and aligning the limited geriatric mental healthcare resources across Sweden.

Furthermore, previous time-trends analyses of depression prevalence overlook considerable heterogeneity in both the clinical presentation and natural course of late-life depression, which can be revealed using a longitudinal perspective. Old-age depression is characterised by a more chronic clinical course, high rates of relapse, and slow remission.^7,8^ Furthermore, in late life, somatic and cognitive symptoms are both common and often present in the absence of core symptoms, such as sadness or anhedonia,^8,9^ leading to many older adults failing to reach the diagnostic threshold of clinical depression.^9^ Importantly, even subsyndromal symptomatology can have considerable detrimental consequences in old age partly due to poor detection and treatment.^10^ Accordingly, in an effort to capture subtle changes over time reflecting subsyndromal symptomatology and natural course variability, several studies have examined longitudinal trajectories of a wide range of depressive symptoms assessed using rating scales.^7,8,11–17^

However, only a few studies have explored temporal trends in longitudinal trajectories of late-life depressive symptoms, particularly in Sweden. One notable exception is a recent multi-country study from the SHARE consortium, which found that, within the Swedish sample, the 1950–60 and 1960–70 birth cohorts exhibited both higher levels and steeper slopes in EURO-D depressive scores than their counterparts born in 1920–1930 and 1930–1940—a contrast accentuated in individuals with multiple chronic conditions (multimorbidity).^18^ While this study gives important first insights into the temporal variation of depressive symptom trajectories in Sweden, further analyses are required using physician-verified symptom scales that are less susceptible to misclassification due to physical disease manifestation in advanced age.^19^ Furthermore, throughout the 20th century, Sweden underwent profound societal transformation, which likely impacted not just multimorbidity but also functional impairment,^20^ loneliness^21^ and education^22^—factors consistently linked to late-life depression.^18,22,23^ Yet, the role of these factors in the temporal variation in depressive symptom trajectories remains unclear.

In this study, we aimed to examine temporal differences in longitudinal trajectories of depressive symptoms of older adults aged 60+ from the population-based Swedish National Study on Aging and Care in Kungsholmen (SNAC-K), central Stockholm. By comparing the evolution of intra-individual depressive symptomatology over 19 years among older adults born up to 40 years apart, this work seeks to expand the understanding of the changing burden of late-life depression – including its possible contributing factors—in Sweden.

## Methods

### Study population

This study is based on individual-level data from the ongoing longitudinal population-based SNAC-K study.^24^ SNAC-K used stratified random sampling across 11 age groups of older adults living in central Stockholm (60, 66, 72, 78, 81, 84, 87, 90, 93, 96 and 99+ years). A total of 3363 individuals (response rate: 73%) participated at baseline (2001–2004). A new sample of 60-year-olds was added in 2010–2013 (n=678, response rate: 66%), as well as two new samples of 81-year-olds (one in 2007–2010 [n=194, response rate: 69%] another in 2013–2016 [n=195, response rate: 57%]). The inclusion of these cohorts allows comparison of individuals of the same age separated in time. Data collection at baseline and during the follow-up was performed by trained professionals and included structured social interviews, clinical examinations (covering both mental and physical health) and evaluations of physical and cognitive functioning. In SNAC-K, follow-up assessments were conducted every 6 years until the age of 78 and every 3 years after the age of 78. Participants were not directly involved in conducting research for this study. However, the SNAC-K data collection team is in close contact with participants and continuously gathers feedback and disseminates key study findings at regularly arranged participant forums. In this specific study, we followed individuals for up to a maximum of 19 years from 2001 to 2004 (wave 1) to 2019–2022 (wave 7). To minimise the impact of COVID, 31 March 2020 was chosen as the end of the study. Out of the 4430 initially enrolled participants, we excluded those with prevalent dementia at baseline (n=265), given a strong reliance of MADRS on individual self-report, which could be impaired in dementia. We further excluded participants without any post-baseline assessment of depressive symptoms (n=1205), resulting in a final analytical sample of 2960 individuals (online supplemental figure S1).

### Depressive symptomatology

We used the 10-item Montgomery and Åsberg Depression Rating Scale (MADRS) to measure depressive symptoms.^25^ The scale is part of the Comprehensive Psychopathological Rating Scale (CPRS), which was used by trained physicians performing the medical interviews. Each of the 10 MADRS items is rated from 0 (no symptoms) to 6 (severe symptoms), resulting in a total score ranging from 0 to 60.^25^ Individual MADRS items and corresponding mean values within the analytical sample are presented in online supplemental table S2. MADRS>6 indicates clinically relevant depressive symptomatology.^26^ MADRS has been shown to have good diagnostic accuracy in populations aged 65 and older.^27^

### Birth cohort

**Figure 1:**
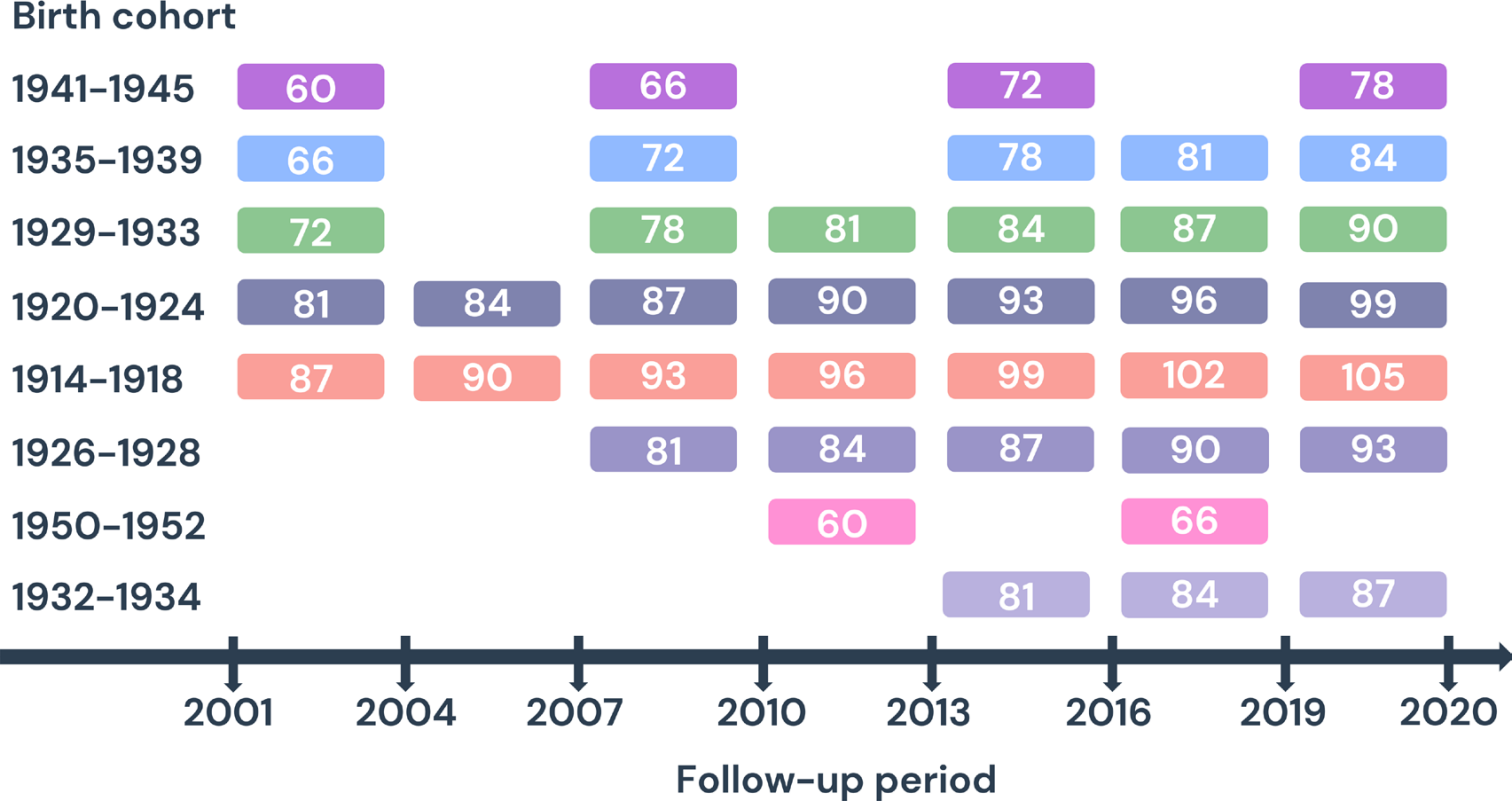
Birth cohorts, ages and follow-up frame in the Swedish National Study of Aging and Care in Kungsholmen (SNAC-K). *Notes:* Relevant cohort ages are displayed inside the boxes for a given year of assessment. Follow-up in SNAC-K is still ongoing. This particular study ended on 31 March 2020, to minimise the impact of the COVID-19 pandemic on depressive symptoms.

In the study population, the oldest person was born in 1904 and the youngest in 1951. We aggregated individuals into eight birth cohort bins. Five underwent baseline assessment in 2001–2004: birth cohorts 1914–1918, 1920–1924, 1929–1933, 1935–1939 and 1941–1945. Additionally, the 1926–1928 birth cohort was assessed for the first time in 2007–2010; the 1950–1952 birth cohort in 2010–2013 and the 1932–1934 birth cohort in 2013–2016 (figure 1). Selection of cohorts was determined by considering sample size, while also aligning the data with age at baseline.

### Depression correlates

Education, functional impairment and loneliness were included as dichotomized covariates. Education was aggregated into primary education vs high school or higher, with the cut-off chosen based on the mean education in the sample. Functional impairment was classified according to the sum score of Activities of Daily Living (ADL) and Instrumental Activities of Daily Living (IADL) categorised as lower and greater functional impairment in relation to the sample mean (ADL+IADL impairments: 0.2, SD=0.9). Feelings of loneliness were categorised as no feelings of loneliness vs experienced feelings of loneliness, based on responses to the Likert-scale question “Do you ever feel lonely?” (1. Yes, often 2. Yes, sometimes 3. No, rarely 4. No, never 5. No, never; responses 1 and 2 indicated loneliness; 3–5 its absence). Smoking, alcohol consumption and use of antidepressants were presented to further describe the sample (see online supplemental table S1) but were not considered in further analyses.

### Statistical analysis

Characteristics of the analytical sample were summarised according to birth cohort and differences were assessed using analysis of variance (ANOVA) for continuous and χ^2^ tests for categorical variables.

Linear mixed-effects models were used to evaluate the change in MADRS scores over 19 years of follow-up. Baseline year, age and birth year (all continuous), as well as sex, were included as fixed effects. To account for possible nonlinear effects, age and birth year were modelled as natural cubic splines (df=2). Interactions between baseline year and age, as well as between birth year and age, were additionally added as fixed effects to explore the differences in MADRS change across the combinations of cohorts and relevant age groups, while accounting for some age-period-specific variation. All models used age as a time scale and included both a random intercept and a random slope to account for individual variability in MADRS baseline levels and rates of change over time. Model selection was based on the Bayesian Information Criterion (BIC), comparing models with up to three df and excluding natural cubic splines for birth year. Survey weights were not applied in the present analyses, as the aim of the study was to estimate age- and cohort-specific longitudinal trajectories of depressive symptoms rather than crude marginal population means or prevalence. SNAC-K recruitment was stratified by age group, and the corresponding design variables relevant to our research question (age, birth year, baseline year and sex) were explicitly incorporated into the mixed-effects models, where age was used as the time scale. Accordingly, the reported estimates should be interpreted as model-based mean trajectories conditional on the covariates included in the model, rather than as weighted marginal estimates for all older adults in Sweden.

**Figure 2:**
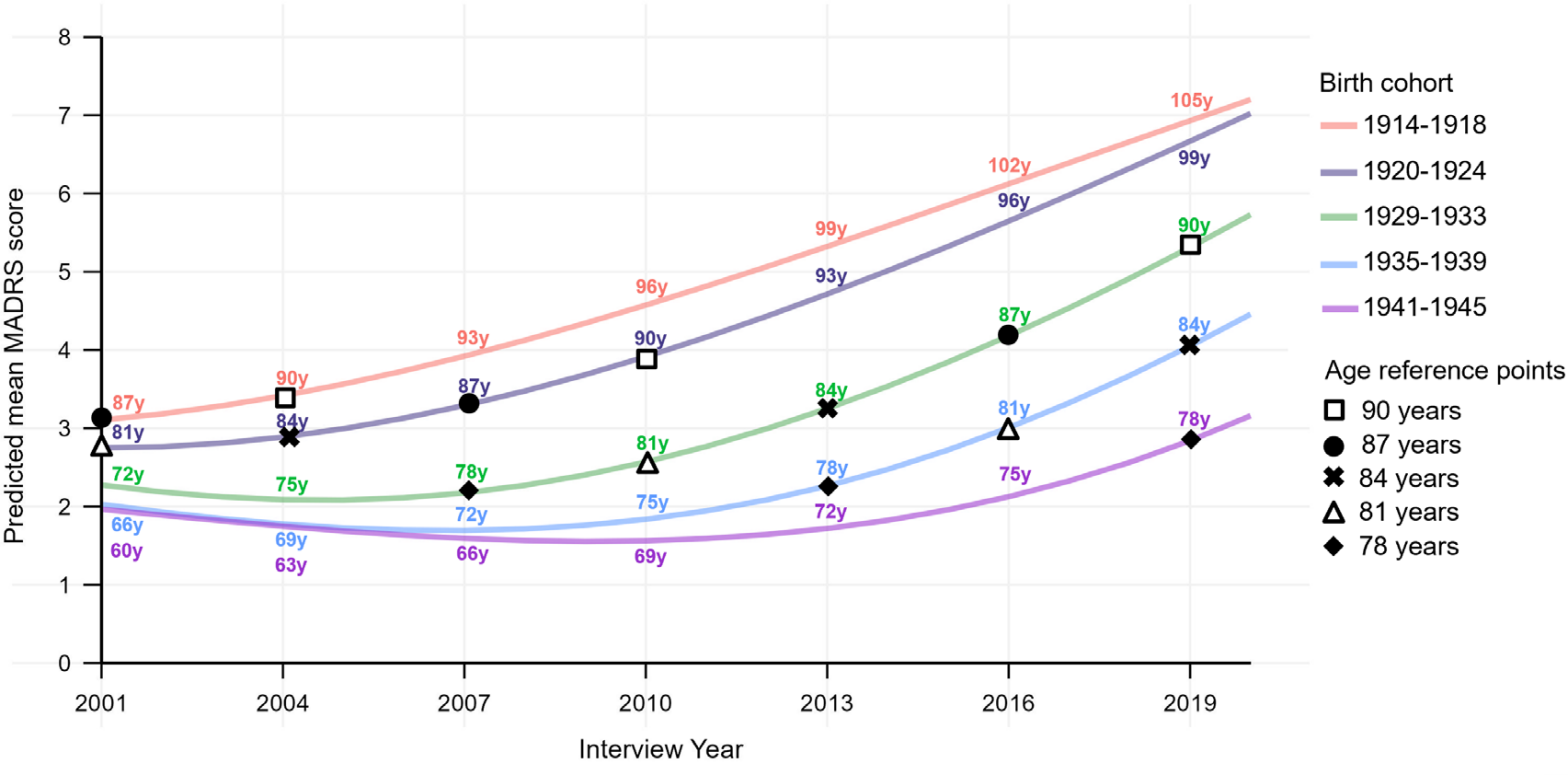
Predicted trajectories of MADRS change by birth cohort during 2001–2020 in the original SNAC-K population with baseline assessment in 2001–2004, N=2169. MADRS, Montgomery and Åsberg Depression Rating Scale; SNAC-K, Swedish National Study on Aging and Care in Kungsholmen.

**Figure 3:**
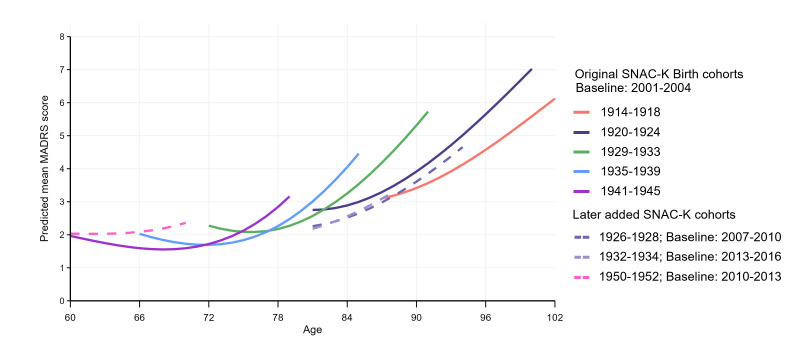
Longitudinal MADRS trajectories by birth cohort across age. Original and later-added SNAC-K cohorts, N=2960. MADRS, Montgomery and Åsberg Depression Rating Scale; SNAC-K, Swedish National Study on Aging and Care in Kungsholmen.

To aid interpretation, predicted MADRS trajectories from the linear mixed-effects model were generated at each year of assessment across unique combinations of birth year and baseline age using the mean values of covariates in the analytical sample (eg., proportion women: 60%). Two sets of predicted MADRS trajectory plots are presented. First, to demonstrate the age-dependent pattern of MADRS accumulation (figure 2), we focused on the subsample of individuals who were all examined during the baseline assessment in 2001–2004 (age range 60–99 years) and were followed for up to 19 years until 2020 (age range 77–106 years). To aid comparison, reference points highlighting MADRS values among individuals of the same age from different birth cohorts assessed at different points in time were added. In figure 3, we additionally included MADRS trajectory slopes for the 60- and 81-year-old individuals assessed for the first time after the 2001–2004 baseline to further examine generational differences. Main plots were presented without uncertainty bounds to facilitate interpretation. In supplementary materials, gradient shading for 95% CIs was added to indicate statistical overlap in predicted MADRS trajectories for the different cohorts (online supplemental figure S2).

**Figure 4:**
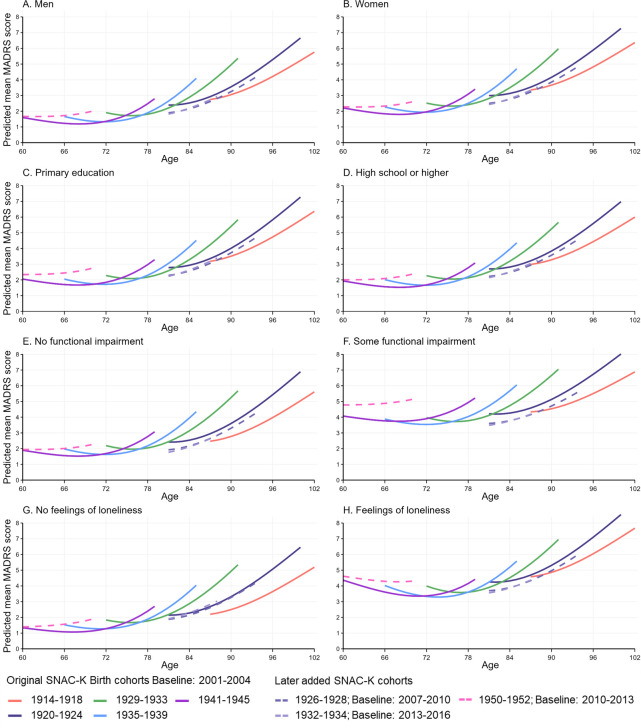
Longitudinal MADRS trajectories by birth cohort across age. Original and later-added SNAC-K cohorts, N=2869. All models were adjusted for birth year, age, baseline year and sex. Adjustment for education, functional impairment and loneliness were performed in separate models. MADRS, Montgomery and Åsberg Depression Rating Scale; SNAC-K, Swedish National Study on Aging and Care in Kungsholmen.

To understand whether depressive trajectories were explained by education, functional impairment and loneliness, analyses were repeated with these factors incorporated as fixed effects, but also as interactions with age and birthyear to explore any trajectory-altering effects. Corresponding MADRS prediction plots were generated at the relevant covariate levels (primary vs secondary/higher education; zero versus some ADL+IADL impairments; no vs some feelings of loneliness), all assessed at baseline (figure 4).

To ensure that the results were not driven by cognitive impairment, analyses were repeated after excluding participants who developed dementia at any point during the follow-up. Furthermore, analyses were repeated after including participants with fewer than two MADRS measurements whose inclusion should not affect MADRS slope estimation but might alter predicted intercept levels. Further to assess non-linearity, quantile regression models were estimated at the lower quartile, median and upper quartile.

## Results

A total of 2960 eligible participants were included in the analytical sample, with a mean age of 70.4 years and 63.2% women. Background characteristics of the eight birth cohorts assessed during their respective baselines are presented in online supplemental table S1.

In all cohorts, most participants were female. The proportion of university-educated individuals increased from 17.3% in earlier-born cohorts (1914–1918) to 67.9% in the later-born ones (1950–1952). While both the proportion of being unpartnered and lonely increased with age, possibly due to an age-related rise in widowhood,^28^ comparing individuals of the same age assessed at different time points revealed only negligible temporal variation in civil status or loneliness. In contrast, the share of current smokers not only declined with age but also became less frequent among the recent birth cohorts. A similar age-related trend was noted for alcohol consumption, although in contrast to declining smoking rates among the recent cohorts, moderate-to-heavy drinking appeared to increase slightly. Antidepressant use exhibited a fluctuating trend with age and no clear temporal variation. All participants from the analytical sample were dementia-free at baseline and had normal cognitive function (MMSE score: 27.8 to 29.4).

### Depressive symptom trajectories over time (by assessment year)

In figure 2, we report predicted longitudinal trajectories of depressive symptoms for older adults belonging to the five original SNAC-K birth cohorts that were each assessed for the first time during the 2001–2004 baseline and were subsequently followed for up to 19 years until 2020. As each of these cohorts represents a distinct age group at baseline, anchoring them to a common baseline year enables the examination of age-specific trajectories of MADRS change during 2001–2020.

Analysis revealed a clear age gradient in depressive symptomatology, with older individuals presenting consistently higher MADRS scores. This gradient was least pronounced at the 2001 baseline, whereby the difference between the lowest predicted MADRS score among the 60-year-old age group (birth cohort 1941–1945) and the highest predicted MADRS score among the 87-year-old age group (birth cohort 1914–1918) was equivalent to 1.2 MADRS points. At the end of the follow-up period in March 2020, the gap between 1914–1918 and 1941–1945 birth cohorts (ie, individuals aged 105 and 78 years) had increased to 4.1 MADRS points.

This change was a result of an age-dependent increase in MADRS which accelerated markedly after age 72. Notably, the pace of MADRS acceleration varied by birth cohorts and appeared especially pronounced among the later-born cohorts that were assessed more recently. For example, among the 90-year-olds, the predicted MADRS score was 3.4 (95% CI 2.9 to 3.9) for the 1914–1918 birth cohort assessed in 2004 and 5.3 (95% CI 5.1 to 5.5) for the 1929–1933 birth cohort assessed in 2019. This pattern was replicated across all age groups (see relevant reference points in figure 2), with MADRS scores being consistently higher among the later-born birth cohorts of similarly aged individuals assessed more recently. Indeed, the 84-year-olds from the 1935–1939 birth cohort (assessed in 2019) had higher depressive symptomatology than the 90-year-olds from the 1920–1924 birth cohort (assessed in 2010).

### Depressive symptom trajectories by age, including new cohorts of 60-year-olds and 81-year-olds

In figure 3, MADRS trajectories are plotted by age, allowing a direct comparison of similarly aged individuals from different birth cohorts. Additionally, we plot MADRS change slopes for three cohorts added after the original SNAC-K baseline of 2001–2004. These comprise two cohorts of 81-year-olds with baseline in 2007–2010 and 2013–2016, and a cohort of 60-year-olds with baseline in 2010–2013.

Figure 3 reaffirms our previous finding that for similarly aged individuals from the original SNAC-K cohort (baseline in 2001–2004), depressive symptomatology was greater among those from the later-born cohorts assessed more recently. This appears to be a result of faster accumulation of depressive symptoms, as later-born cohorts’ MADRS slopes consistently steepened relative to their earlier-born counterparts at comparable ages (with many contrasts also being statistically significant; see online supplemental figure S2 for details). While restricted follow-up length for the 1935–1939 and the 1941–1945 cohorts prevents an outright comparison with earlier-born cohorts across the entire age-span, the current trend is indicative of late-life MADRS trajectories having accelerated more recently.

This trend was further confirmed when examining MADRS change trajectories in the later-added age group of 60-year-olds from the 1950–1952 birth cohort assessed for the first time in 2010–2013. While having a virtually identical level of depressive symptoms as a similar aged earlier-born cohort from 1941 to 1945 (assessed in 2001–2004), after 10 years, the later-born cohort’s MADRS score increased by 70% more (although the relatively short follow-up yielded wide CIs for this comparison; see online supplemental figure S2 for detail).

This trend, however, was not replicated when additional age groups of 81-year-olds from the 1926–1928 birth cohort (assessed in 2007–2010) and the 1932–1934 birth cohort (assessed in 2013–2016) were introduced. The two later-born cohorts presented with initially lower MADRS levels compared with their similarly aged counterparts from an earlier birth cohort (1920–1924; assessed in 2001–2004). While we noted that MADRS accumulation in the later-born cohorts accelerated—by age 87 outpacing the pace of MADRS change in the earlier-born cohort—restricted follow-up of later-added cohorts of 81-year-olds prevents complete age-span comparison.

Accounting for education, functional impairment, and loneliness did not alter the pattern of estimated depressive symptom trajectories. Examining predicted MADRS slopes at the relevant covariate contrasts (figure 4) indicated (1) marginally higher symptom levels in women than in men; (2) virtually no differences across educational groups; (3) higher depressive burden in older adults with functional impairment; and (4) higher depressive burden in those reporting feelings of loneliness.

Finally, results remained unchanged after excluding participants with incident dementia, although slightly lower MADRS levels were observed for the earlier-born cohorts (online supplemental figure S3). Similarly, the overall pattern was preserved when the analysis was repeated including participants with fewer than two MADRS measures, although mean MADRS levels were marginally higher in this sample. Findings from the quantile regression models were also consistent with the overall trend.

## Discussion

In this population-based analyses of 19-year depressive trajectories of older adults born up to 40 years apart, we observed two main findings: first, an age-related acceleration of depressive symptomatology that appears to steepen around age 72 and, second, an increasing burden of depressive symptoms among the later-born cohorts assessed more recently. These patterns persisted after accounting for known sociodemographic and functional correlates of depression. Our findings from the SNAC-K study in central Stockholm suggest that despite some previous studies suggesting improvement based on prevalence measures, when assessed through longitudinal symptom change, old-age depressive burden may indeed be increasing with time, which, given the rapid ageing of the Swedish population, demands attention from relevant stakeholders.

Our findings highlight the importance of age in the evolution of depressive symptoms, as across all birth cohorts, symptomatology increased as people became older. This is consistent with previous multi-country studies showing symptom acceleration after age 70.^18,29^ Notably, mean predicted depressive symptom levels among centenarians in our data were comparable to those associated with mild depression,^30^ underscoring the need for mental health monitoring in older individuals often facing multiple comorbidities.^31–34^ Clinically relevant levels of depressive symptoms, found here among the very old, also align with previous reports of an age-related increase in the burden of late-life depressive disorders both globally^35^ and in the Nordics,^36^ including Sweden, based on both rating scales^4,37^ and register-based data.^38^ Our study advances this work with an important longitudinal perspective leveraging intraindividual change in depressive symptomatology. Accelerated accrual of depressive symptoms with age likely reflects biological and psychosocial changes associated with late life – such as physical health deterioration, social role transitions, and bereavement.^31,39,40^ Critically, older adults in Sweden continue to face limited access to psychological therapies and fragmented care pathways, with considerable regional variation.^2,41^ They are also less likely than younger adults to receive specialist psychiatric care or psychotherapy and are more often treated within primary care,^41^ suggesting that current care systems may not be fully adapted to the complex mental health needs of an ageing population.

Our study demonstrates that at comparable ages, later-born cohorts in SNAC-K appear to have experienced an increasing burden of late-life depressive symptoms relative to their earlier-born counterparts. This was evident in the analysis of the original SNAC-K cohort followed since 2001 but also after considering refresher cohorts of 60-year-olds recruited in 2010–2013. On the one hand, this contradicts two repeated cross-sectional studies from Gothenburg reporting a decline over time among the 70-year-olds (between 1976 and 2016)^6^ and 85-year-olds (throughout 1986, 2008, and 2015).^5^ Discrepancy in findings is likely due to methodological differences, including a wider age span and longitudinal perspective on symptom change employed here. On the other hand, our findings align with recent analyses of the Swedish subset of the SHARE data which modelled depressive trajectories,^18,29^ as well as a study in Northern Sweden that reported higher prevalence of depressive disorders among the 90- and 95-year-olds born 15 years apart.^4^ Furthermore, analysis of trends in mid-life depression can help clarify the patterns in older age. Thus, a previous study has found that later-born 18–29-year-olds from Sweden appear to exhibit a greater burden, as reflected in increased self-reported psychological symptoms, more treatment and more psychiatric service use.^42,43^ More broadly, a growing body of evidence indicates that trends of depression and anxiety have increased across younger as well as older adults across different countries.^44–46^ Still, more evidence is needed for reliable patterns to emerge, and our own finding of no increase in symptoms among the refresher cohorts of 81-year-olds underscores this uncertainty. Further work that includes a wider range of Swedish regions and spans extended time periods is needed to verify these trends.

Faster accrual of depressive symptoms among later-born cohorts in our data suggests that the experience of ageing differs across time periods and generations. Although late-life health has improved on some metrics—for example, cognitive performance and cardiovascular outcomes (especially stroke)—these gains have primarily extended life expectancy rather than reduced years lived with disability.^47,48^ Furthermore, recent studies from Sweden suggest that later-born cohorts increasingly survive into higher ages with complex chronic disease patterns or frailty.^49–51^ Such increases in health complexity could contribute to depressive symptomatology, given the strong bidirectional association between somatic disease burden and depression in late life.^52,53^ Furthermore, while the later-born cohorts likely experienced greater advancements in education, living standards and medical care, mental health awareness and antidepressant availability,^54,55^ psychosocial factors such as loss of purpose and autonomy, rapid social changes associated with digitalisation and social isolation^3,56–58^ may have attenuated these gains. Finally, age-specific variation possibly explains why we noted a reversal of the general trend among the 81-year-olds, but not the 60-year-olds assessed more recently.

The persistence of age- and cohort-related patterns after adjusting for education, functional limitations and loneliness highlights robust temporal trends, although symptom levels differed across covariate strata. For example, contrary to previous studies,^22^ we did not observe differences in symptom levels over time by education. This may indicate a reduced influence of education on depressive symptoms in the more recent cohorts, potentially due to increased educational attainment or broader access to health and social resources.^59–61^ Furthermore, our findings show that individuals with functional limitations and loneliness had higher levels of depressive symptoms while cohort patterns remained, which is in line with previous research showing higher but consistent cohort patterns for multimorbidity^18^ and relatively stable loneliness trends over time in Sweden.^21^ We did not adjust the analysis for antidepressant use, as it is unlikely to serve as a confounder (ie, common cause of exposure and outcome) in the association between birth cohort and depressive symptom changes. However, therapeutically suppressed depressive burden in individuals with antidepressants could have affected MADRS trends if antidepressant use itself has changed over time. While we only noted a minor increase in our data (see online supplemental table S1), for Sweden as a whole, the prevalence of antidepressant use has increased in all age groups by 34% between 2006 and 2021 and was highest in individuals aged 65 years or older based on data from the prescription registers.^62^ This increase likely reflects rising prevalence and recognition of depression and anxiety; guideline changes recommending antidepressants as first-line treatment for anxiety; as well as wider availability of newer antidepressants with improved risk-benefit profiles and expanded indications beyond depression (eg., sleep disorders, chronic pain). Therefore, in light of these trends in antidepressant use, our finding of steeper depressive symptom trajectories in recent birth cohorts could be viewed as an underestimation of the true burden in these groups. We encourage future work, possibly using the dual change framework, to examine concurrent temporal trends in antidepressant use and depressive symptoms.

### Strengths and limitations

The strengths of this study include its population-based design, a long follow-up period of 19 years, repeated and thorough assessments of depressive symptoms and high response rates. Reporting bias was further mitigated by psychiatric assessment performed by trained physicians. However, some limitations should be noted. First, selection bias could be introduced by longitudinal attrition, as participants required at least two MADRS measurements to be included in the analysis. However, this is largely unavoidable in ageing cohorts and SNAC-K has high retention rates through extensive outreach efforts and geographic proximity. Second, selective survival could contribute to the underestimation of MADRS trajectories, especially later in life, as individuals with lower MADRS scores may be more likely to survive into higher ages. Over time, however, the share of individuals surviving with previously lethal conditions has likely increased, which could accentuate the seemingly worsening of the mental health burden in recent cohorts. Therefore, our results should be interpreted in the context of the possible compositional differences between the cohorts. Third, given how depressive symptoms may be due to incipient dementia, we excluded prevalent cases of dementia at baseline and further dropped incident dementia cases during follow-up in sensitivity analyses to mitigate potential confounding. Finally, SNAC-K consists of an urban and relatively affluent population, which limits generalisability to similar settings and may lead to an underestimation of depressive symptom burden relative to less advantaged populations. At the same time, repeated physician-based assessments and detailed follow-up support strong internal validity. Accordingly, our estimates are best interpreted as model-based longitudinal trajectories in the SNAC-K source population rather than as crude weighted population estimates for Sweden as a whole.

## Conclusions

This population-based study from the SNAC-K study has shown that depressive symptomatology accelerates with age and is greater among the later-born birth cohorts of older adults in central Stockholm. Together, these findings suggest that the depressive burden among Swedish older adults may be increasing, which—given the demographic shifts—represents a significant public health challenge. Mental healthcare needs to be adapted using an age perspective that recognises the unique challenges of ageing for prevention, treatment, and management of depression.

## Supplementary material

10.1136/bmjph-2026-005098 online supplemental file 1

## Data Availability

Data may be obtained from a third party and are not publicly available.
