# Supplementary material for: Temporal trends in late-life depressive symptom trajectories: a longitudinal population-based cohort study from Stockholm, Sweden
Source: BMJ Public Health. 2026 Jul 22;4(3):e005098. doi: 10.1136/bmjph-2026-005098 (PMC13404410; doi:10.1136/bmjph-2026-005098)
Supplement: online supplemental file 1 [file bmjph-4-3-s001.pdf]

Supplementary material

Table S1. Baseline descriptive characteristics of the study population by birth cohort and year of baseline.

Baseline year	OVERALL	2001-2004					2007-2010	2013-2016	2010-2013	
**Birth cohort & age group at baseline**		**1914-1918** 87+ years	**1920-1924** 81 years	**1929-1933** 72 years	**1935-1939** 66 years	**1941-1945** 60 years	**1926-1928** 81 years	**1932-1934** 81 years	**1950-1952** 60 years	
	**N=2,960**	**N=330**	**N=479**	**N=329**	**N=431**	**N=600**	**N=124**	**N=132**	**N=535**	**p-value**
**Age**	70.4 (±10.0)	87.7 (±3.6)	79.1 (±1.5)	72.1 (±0.4)	66.1 (±0.3)	60.4 (±0.5)	81.2 (±0.4)	81.1 (±0.3)	60.2 (±0.4)	<0.001
**Sex**										
Man	1,090 (36.8%)	83 (25.2%)	152 (31.7%)	124 (37.7%)	171 (39.7%)	263 (43.8%)	34 (27.4%)	44 (33.3%)	219 (40.9%)	<0.001
Woman	1,870 (63.2%)	247 (74.8%)	327 (68.3%)	205 (62.3%)	260 (60.3%)	337 (56.2%)	90 (72.6%)	88 (66.7%)	316 (59.1%)	
**Education**										
Elementary	306 (10.3%)	90 (27.4%)	88 (18.4%)	39 (11.9%)	28 (6.5%)	33 (5.5%)	21 (16.9%)	7 (5.3%)	0 (0.0%)	<0.001
High school	1,354 (45.8%)	182 (55.3%)	266 (55.5%)	174 (52.9%)	206 (47.8%)	230 (38.3%)	63 (50.8%)	61 (46.2%)	172 (32.1%)	
University	1,299 (43.9%)	57 (17.3%)	125 (26.1%)	116 (35.3%)	197 (45.7%)	337 (56.2%)	40 (32.3%)	64 (48.5%)	363 (67.9%)	
**Civil status**										
Partnered	1,591 (53.8%)	70 (21.3%)	198 (41.3%)	187 (56.8%)	271 (62.9%)	380 (63.4%)	46 (37.1%)	57 (43.2%)	382 (71.4%)	<0.001
Unpartnered	1,367 (46.2%)	259 (78.7%)	281 (58.7%)	142 (43.2%)	160 (37.1%)	219 (36.6%)	78 (62.9%)	75 (56.8%)	153 (28.6%)	
**Alcohol**										
No or occasional	763 (25.9%)	172 (52.4%)	178 (37.3%)	84 (25.7%)	95 (22.1%)	86 (14.3%)	36 (29.3%)	38 (28.8%)	74 (13.9%)	<0.001
Moderate to heavy	2,186 (74.1%)	156 (47.6%)	299 (62.7%)	243 (74.3%)	334 (77.9%)	514 (85.7%)	87 (70.7%)	94 (71.2%)	459 (86.1%)	
**Smoking**										
Never/ever	2,558 (87.0%)	303 (92.4%)	433 (90.6%)	283 (86.8%)	370 (86.4%)	477 (80.0%)	113 (91.9%)	124 (95.4%)	455 (85.8%)	<0.001
Current	381 (13.0%)	25 (7.6%)	45 (9.4%)	43 (13.2%)	58 (13.6%)	119 (20.0%)	10 (8.1%)	6 (4.6%)	75 (14.2%)	
**Loneliness**										
No	2,285 (77.9%)	212 (65.2%)	361 (76.0%)	255 (78.9%)	345 (80.8%)	475 (79.8%)	98 (79.0%)	107 (81.7%)	432 (80.9%)	<0.001
Yes	649 (22.1%)	113 (34.8%)	114 (24.0%)	68 (21.1%)	82 (19.2%)	120 (20.2%)	26 (21.0%)	24 (18.3%)	102 (19.1%)	
**MADRS score**	2.3 (±3.6)	3.1 (±4.0)	2.9 (±3.9)	1.9 (±3.0)	2.0 (±3.3)	2.1 (±3.5)	1.8 (±3.6)	2.2 (±3.1)	2.0 (±3.9)	<0.001
**Use of antidepressants**										
No	2,730 (92.3%)	300 (90.9%)	436 (91.0%)	303 (92.1%)	411 (95.4%)	555 (92.5%)	114 (91.9%)	120 (90.9%)	491 (91.9%)	0.32
Yes	229 (7.7%)	30 (9.1%)	43 (9.0%)	26 (7.9%)	20 (4.6%)	45 (7.5%)	10 (8.1%)	12 (9.1%)	43 (8.1%)	
**MMSE score**	28.9 (±1.7)	27.8 (±1.9)	28.7 (±1.5)	29.1 (±1.0)	29.4 (±0.9)	29.4 (±1.3)	28.3 (±1.8)	28.4 (±1.5)	29.0 (±2.4)	<0.001
**ADL + IADL**	0.2 (±0.9)	1.0 (±1.8)	0.3 (±0.9)	0.1 (±0.4)	0.0 (±0.3)	0.0 (±0.5)	0.4 (±1.0)	0.4 (±0.9)	0.0 (±0.3)	<0.001

Data are presented as mean ± standard deviation or number (proportion, %). A color gradient was added to facilitate comparison of similarly aged individuals belonging to different birth cohorts assessed at different SNAC-K baselines.

Abbreviations: MADRS, Montgomery and Åsberg Depression Rating Scale; MMSE, Mini-Mental State Examination; ADL, Activities of Daily Living; IADL, Instrumental Activities of Daily Living.

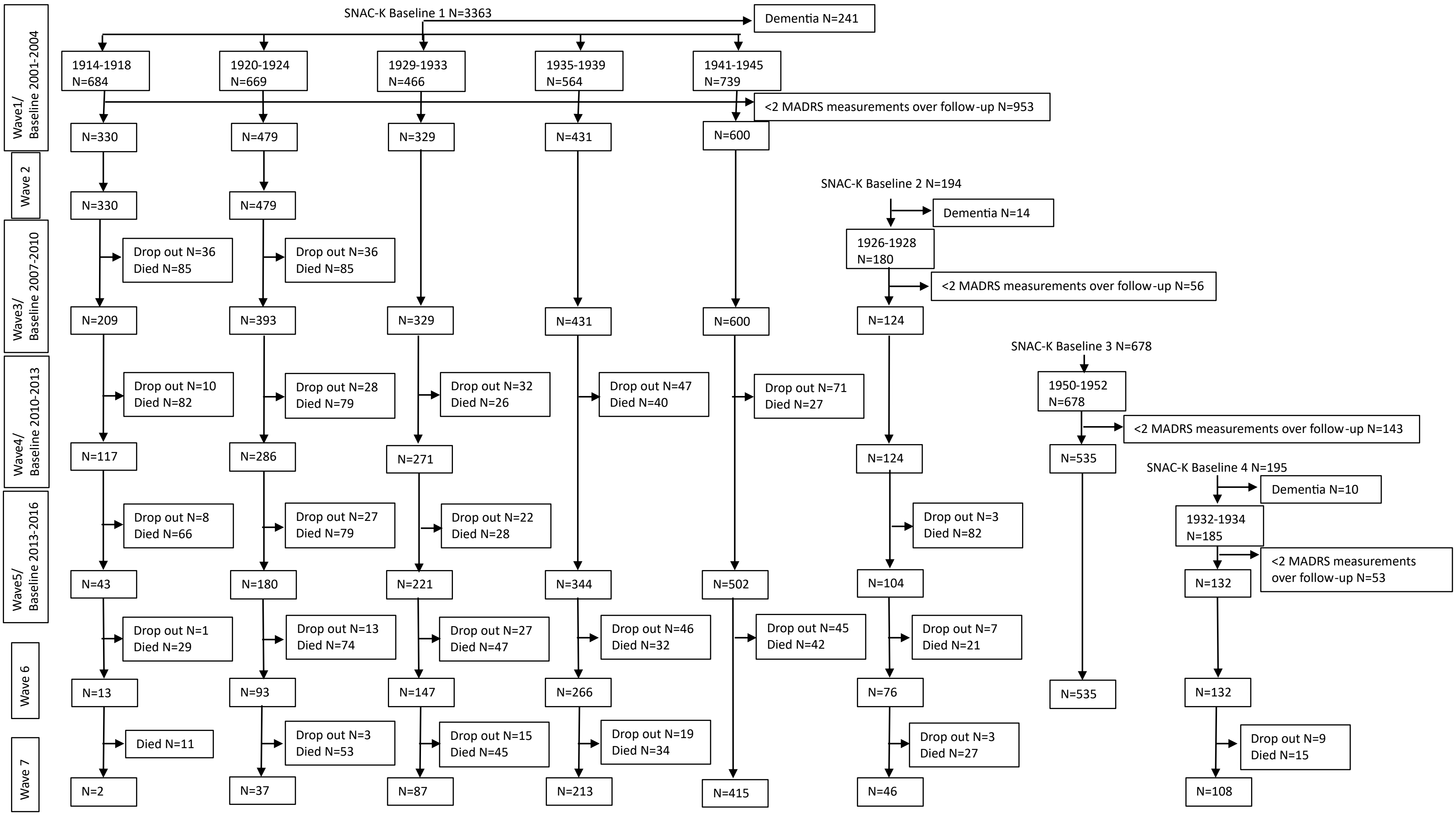

Figure S1. Flow-chart of the study population

Table S2. Mean levels of 10 individual depressive symptoms included in MADRS at baseline

Item list		Mean score	SD
1.	Apparent sadness	0.1	±0.5
2.	Reported sadness	0.2	±0.5
3.	Inner tension	0.3	±0.8
4.	Reduced sleep	0.5	±1.0
5.	Reduced appetite	0.1	±0.6
6.	Concentration difficulties	0.3	±0.8
7.	Lassitude	0.4	±0.8
8.	Inability to feel	0.1	±0.4
9.	Pessimistic thoughts	0.2	±0.6
10.	Suicidal thoughts	0.1	±0.4

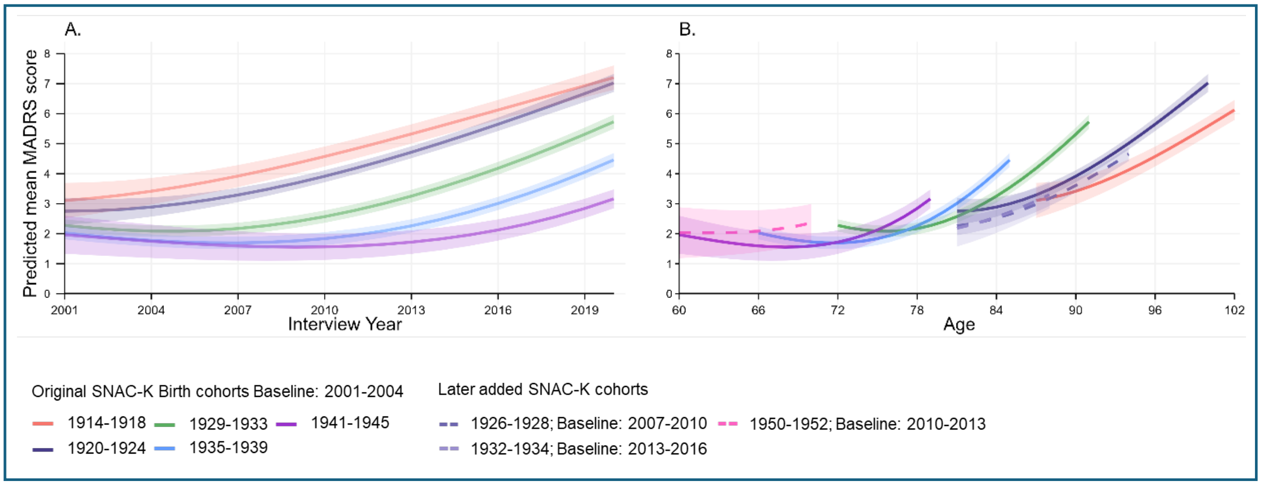

Figure S2. Longitudinal MADRS trajectories with confidence intervals by birth cohort: (A) across follow-up time 2001-2020 in the original SNAC-K population with baseline assessment in 2001-2004 (N=2169); and (B) by age, including original and later-added SNAC-K cohorts.

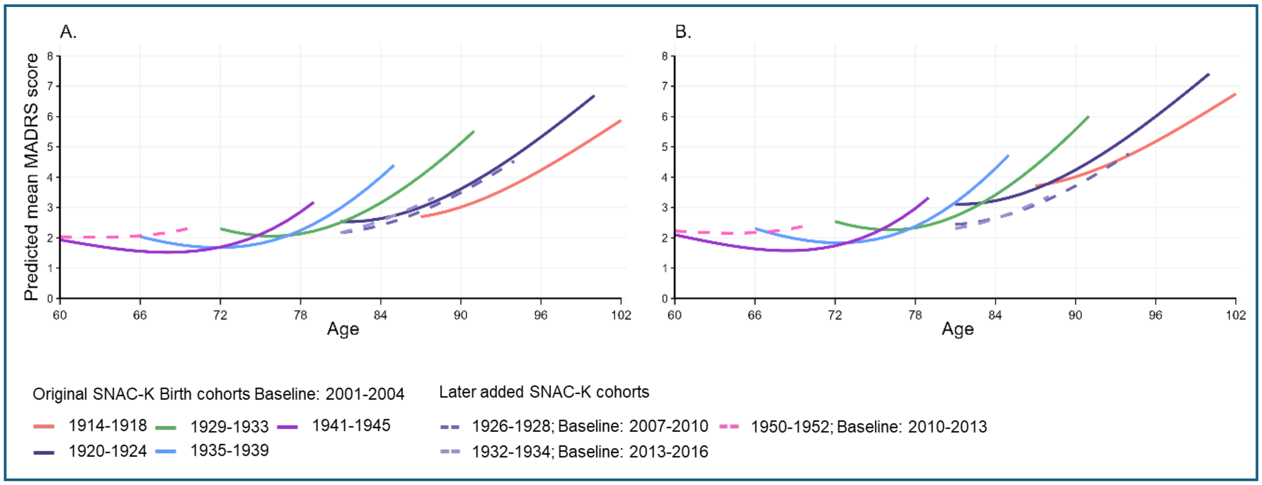

Figure S3. Longitudinal MADRS trajectories by birth cohort across age. Original and later-added SNAC-K cohorts. (A) Excluding participants with incident dementia (N=2498). (B) Including participants with less than two MADRS measures (N=4071).
